# Recurrent posterior fossa group A (PFA) ependymoma in a young child with constitutional mismatch repair deficiency (CMMRD)

**DOI:** 10.1111/nan.12862

**Published:** 2022-11-18

**Authors:** Mayen Briggs, Anirban Das, Helen Firth, Adrian Levine, Santiago Sánchez‐Ramírez, Logine Negm, Ayse B. Ercan, Jill Chung, Vanessa Bianchi, Ibrahim Jalloh, Poe Phyu, Nicky Thorp, Richard G. Grundy, Cynthia Hawkins, Jamie Trotman, Patrick Tarpey, Uri Tabori, Kieren Allinson, Matthew J. Murray, Genomics England Research Consortium

**Affiliations:** ^1^ Department of Neuropathology Cambridge University Hospitals NHS Foundation Trust Cambridge UK; ^2^ The International Replication Repair Deficiency Consortium (IRRDC), Division of Haematology/Oncology The Hospital for Sick Children Toronto ON Canada; ^3^ Department of Genetics Cambridge University Hospitals NHS Foundation Trust Cambridge UK; ^4^ Department of Neurosurgery Cambridge University Hospitals NHS Foundation Trust Cambridge UK; ^5^ Department of Radiology Cambridge University Hospitals NHS Foundation Trust Cambridge UK; ^6^ Department of Radiation Oncology The Christie Proton Beam Therapy Centre Manchester UK; ^7^ Children's Brain Tumour Research Centre, Biodiscovery Unit University of Nottingham Nottingham UK; ^8^ Division of Neuropathology The Hospital for Sick Children Toronto ON Canada; ^9^ East‐Genomics Laboratory Hub (GLH) Genetics Laboratory Cambridge University Hospitals NHS Foundation Trust Cambridge UK; ^10^ Department of Pathology University of Cambridge Cambridge UK; ^11^ Department of Paediatric Haematology and Oncology Cambridge University Hospitals NHS Foundation Trust Cambridge UK

**Keywords:** CMMRD, congenital mismatch repair deficiency, ependymoma, Lynch syndrome, PMS2

**Key Points**

- We present an unusual case of a posterior fossa group A (PFA) ependymoma in a 17‐month‐old child with café‐au‐lait macules but negative for neurofibromatosis 1 (NF1).
- Whole genome and targeted resequencing of paired tumour/normal DNA revealed biallelic pathogenic germline variants in *PMS2*, consistent with the clinical diagnosis of CMMRD.
- Through international collaboration, we describe the genomic profile of both the primary and recurrent tumours, confirming the ependymoma has indeed arisen from DNA replication defects.
- PFA ependymoma has not previously been reported in CMMRD, and given the significant management implications for the patient and at‐risk family members, screening for CMMRD should be strongly considered in all malignant paediatric CNS neoplasms.

Constitutional mismatch repair deficiency (CMMRD) is a hereditary cancer syndrome, characterised by biallelic germline pathogenic variants in one of the four DNA mismatch repair (MMR) genes, mutS homologue 2 (*MSH2*), mutS homologue 6 (*MSH6*), mutL homologue 1 (*MLH1*), or postmeiotic segregation increased 2 (*PMS2*) [1]. First described in 1999 [2, 3] and initially considered rare, there have been increasing reports of this syndrome over the past decade, and it is now considered as being underdiagnosed, particularly with higher prevalence among endogamous populations [4] and in developing countries [5]. Individuals with CMMRD are at high risk of developing multiple malignancies across the central nervous system (CNS), haematological, and gastrointestinal systems, often with cutaneous features mimicking neurofibromatosis type 1 (NF1) [6]. Cancers develop in childhood, with CNS malignancies presenting at a mean age of 8 years [7], with reports of haematological malignancies manifesting in patients as young as 12 months [2]. These cancers are unusually aggressive and often rapidly fatal after failing chemotherapy and radiotherapy. However, recent reports suggest remarkable responses and prolonged survival following PD1‐immune checkpoint inhibition, driven by their elevated mutation burden and genome‐wide microsatellite instability (MSI) [8]. Accurate and early diagnosis is therefore of paramount importance, as in addition to opportunities to use appropriate immune‐directed therapies, cascade testing and surveillance have been recently demonstrated to profoundly impact survival, both for the individual and the family [6, 8]. CMMRD‐associated malignant brain tumours include high‐grade‐gliomas, medulloblastoma and other embryonal tumours [9]. Ependymomas have not previously been described in the context of CMMRD. Here, we present a case of a young child undergoing evaluation for café‐au‐lait macules who developed an ependymoma and was subsequently confirmed to have CMMRD. We describe the extensive collaborative genomic analyses performed to confirm this. We emphasise pertinent ‘real‐world’ implications, as these findings directly impacted patient management, specifically the use of immunotherapy at second relapse, after the failure of conventional chemo‐radiation approaches.

A 17‐month‐old female, known to the genetics team due to the presence of multiple pre‐existing café‐au‐lait macules, presented with vomiting and lethargy. Targeted sequencing had failed to identify any pathogenic germline variants in *NF1* (*neurofibromin‐1*) or *SPRED1* (*Sprouty‐related, EVH1 domain‐containing‐protein‐1*), and clinically, she did not fulfil NF1 criteria (as expected, given her young age). Her parents were nonconsanguineous, with no significant family history of cancer. Head MRI scan showed a heterogeneously enhancing, solid and cystic mass within the fourth ventricle extending to the medulla (Figure 1A), but no other disease. Complete gross‐total‐resection (GTR) was performed, confirmed on postoperative imaging, and histological examination of the resected tumour specimen confirmed an ependymoma (Figure 1B) with a Ki67 index of 12% (Figure 1C). Cerebrospinal fluid (CSF) cytology was negative. Molecular subgrouping using immunohistochemistry suggested that this was likely a posterior fossa group A (PFA) ependymoma, based on loss of expression of H3K27me3 (Figure 1D) and strong expression of EZHIP (Figure 1E). Methylation profiling further confirmed this as a PFA ependymoma (DKFZ classifier v12.5, calibration score: 0.99) with no high‐risk cytogenetic aberrations, such as 1q gain or 6q loss [10] (Figure 1F,G). The diagnosis of a malignant brain tumour, in association with café‐au‐lait macules, but in the absence of *NF1/SPRED1* germline variants, raised the possibility of neurofibromatosis type 2 (NF2) or CMMRD and warranted further investigation. Further immunohistochemical analysis was requested directed against the four MMR proteins in both tumour and nontumour tissue. The tumour showed retained MLH1, MSH2 and MSH6 expression but loss of PMS2 expression in both neoplastic cells and background normal brain tissue (Figure 1H). Functional testing for genomic microsatellite indel (MSI) accumulation, through low‐pass genome sequencing at the International Replication‐Repair Deficiency Consortium (www.replicationrepair.ca), corroborated high genomic MSI matching other known MMR‐deficient brain tumours (Figure 1I). Targeted sequencing subsequently identified two germline pathogenic variants in *PMS2*, a heterozygous frameshift variant c.1831dupA p.(lle611Asnfs*2) inherited from the mother (age: 34 years), and a heterozygous deletion of exon 12 inherited from the father (age: 44 years), thereby confirming the clinical diagnosis of CMMRD. The first variant, located in exon 11 of the *PMS2* gene, is predicted to cause a truncated or absent protein due to a translational frameshift and has been reported in multiple patients with Lynch syndrome [11, 12] and children with CMMRD, including an 11‐year‐old with high‐grade glioma and osteosarcoma [9], a 14‐year‐old with colorectal adenocarcinoma [13] and a 7‐year‐old with glioblastoma and T‐cell lymphoma [14]. The second variant, on the other allele, contributed to the complete loss of protein expression and has been previously reported in an 11‐month‐old infant with CMMRD presenting with medulloblastoma [15] and a 17‐year‐old with Lynch syndrome presenting with colorectal carcinoma and phenotypically mimicking CMMRD [16].

**FIGURE 1:**
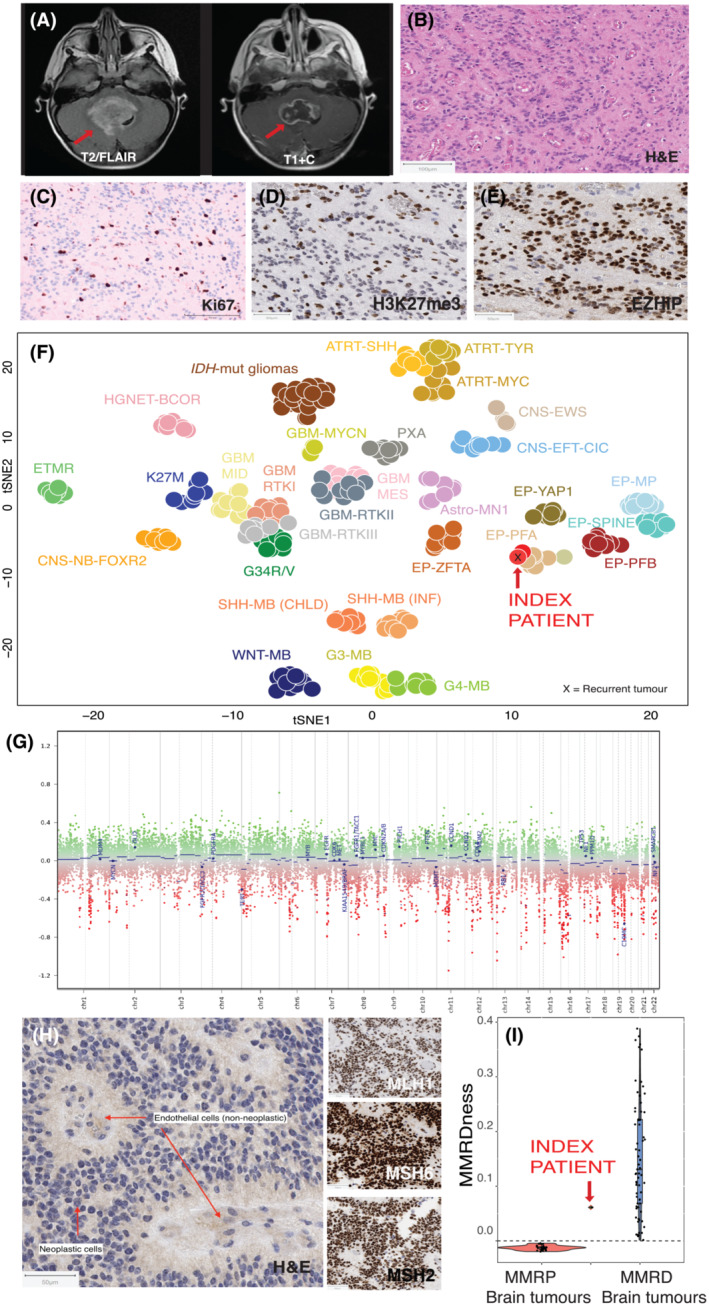
(A) Representative MRI images at first presentation, with the tumour shown with a red arrow using T2/FLAIR and T1 + contrast sequences. (B) Representative histopathology at diagnosis. Haematoxylin and eosin (H&E)‐stained sections from the primary tumour showed classic ependymoma histology, with monotonous populations of tumour cells with round to oval nuclei, fine chromatin, moderate eosinophilic cytoplasm and numerous perivascular anuclear zones (pseudorosettes). (C) The Ki67 labelling index was 12%. (D) Immunohistochemistry for H3K27me3 showed loss of expression in tumour cells, and (E) EZHIP was strongly expressed, confirming a posterior fossa group A ependymoma. (F) A methylation array was performed, followed by unsupervised clustering using t‐distributed‐stochastic‐neighbour‐embedding (tSNE) analysis with previously published molecular subgroups of brain tumours and ependymomas, confirming the diagnosis of the index case (red arrow) as a posterior fossa ependymoma, group PFA. The case was further run through the DKFZ classifier v12.5, which corroborated this diagnosis with a calibrated score of 0.99 (cut‐off for diagnosis >0.9). Controls for tSNE analysis were downloaded from NCBI Gene Expression Omnibus (GEO), accession number GSE109381. (G) Copy number analysis did not reveal gain in chromosome 1q or loss of chromosome 6q. Notably, there were no aberrations detected on either chromosome 17 or 22. (H) Immunohistochemical analysis revealed loss of staining for PMS2 both in the tumour cells and surrounding normal cells, suggesting germline biallelic loss‐of‐function while staining for MLH1, MSH6 and MSH2 were retained both in tumour and normal tissues. (I) Functional characterisation of genomic microsatellite indel accumulation using low‐pass (1X) whole genome sequencing showed ‘MMRD‐ness’ beyond the threshold of ‘zero’, similar to other known CMMRD brain tumours and higher than MMR‐proficient (MMRP) brain tumours. Controls (*n* = 98) for low‐pass genomic microsatellite instability characterisation (LOGIC) were obtained from IRRDC (https://replicationrepair.ca/) and performed according to previously published methods.

The patient was enrolled in the SIOP‐Ependymoma‐II trial and randomised to receive seven ‘standard‐of‐care’ alternating chemotherapy cycles lasting a total of approximately 1 year, involving vincristine, carboplatin, methotrexate, cyclophosphamide, and cisplatin, plus the experimental agent sodium valproate. Surveillance MRI head/spine imaging was continued three‐monthly.

Six months following the commencement of chemotherapy, she presented with asymptomatic localised recurrence, with two new 5‐mm diameter nodules within the primary tumour bed (Figure 2A), and GTR was performed for the second time. Again, there was no evidence of dissemination by imaging or CSF cytology. The recurrent tumour displayed more high‐grade features, such as elevated mitotic activity and microvascular proliferation at recurrence (Figure 2B,C) and a higher Ki67 index (23%), as compared with baseline (12%) (Figure 1B,C). Methylation profiling confirmed the recurrent tumour classified as PFA ependymoma (DKFZ classifier v12.5; calibration score: 0.99) with a balanced copy number profile, as at diagnosis (Figure 1F). She received focal proton radiotherapy to the tumour bed to a total dose of 54Gy in 30 fractions over 6 weeks and was transferred onto the trial observational arm. The delivered 54Gy dose was lower than the standard‐of‐care (59.4Gy in 33 fractions) but was agreed in view of her young age, two previous operations, known CMMRD and prior chemotherapy. Unfortunately, a second asymptomatic localised recurrence was noted on surveillance imaging as a small nodule of enhancing disease after 21 months of follow‐up. A third GTR was performed and histology confirmed this to be recurrent ependymoma (Figure 1E). However, additional analyses were not feasible in view of the limited availability of tissue.

**FIGURE 2:**
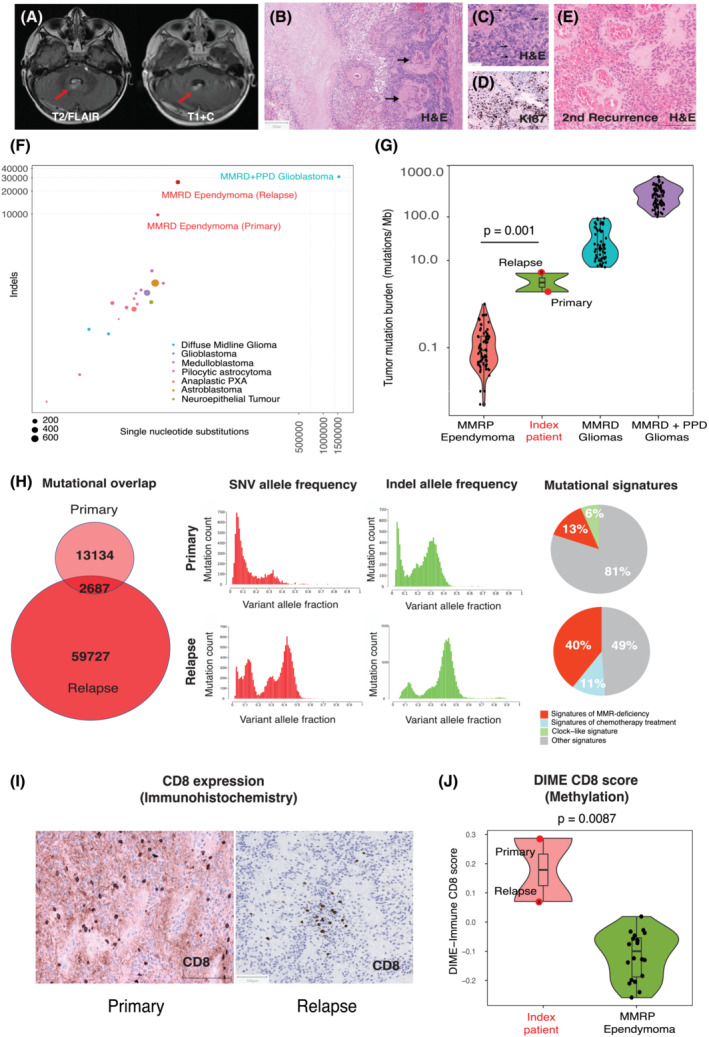
(A) Representative MRI images at recurrence, with the tumour shown with a red arrow using T2/FLAIR and T1 + contrast sequences. (B) The recurrent tumour showed clear anaplastic features, including areas of microvascular proliferation and necrosis along with higher cell density, confluent necrosis and microvascular proliferation (black arrows). (C) The recurrent tumour also showed a higher rate of mitosis (arrows; >35 per 0.24 mm^2^, in comparison with <2 per 0.24 mm^2^ at first resection). (D) The Ki67 index was elevated (23%) (E) Morphology of the tumour at second recurrence, similar to the previous resection, showed anaplastic features with foci of necrosis, apoptotic bodies and notable mitotic activity. (F) High single nucleotide variants and indels both at baseline and recurrence compared with known MMR‐proficient (MMRP) paediatric brain tumours and known CMMRD‐associated glioblastoma (with additional somatic polymerase‐proofreading deficiency; PPD). Controls are from local cases sequenced as part of the NHS‐England 100,000 Genomes Project (https://www.genomicsengland.co.uk/initiatives/100000‐genomes‐project). [Diffuse midline glioma *n* = 2, glioblastoma *n* = 1, medulloblastoma *n* = 5, pilocytic astrocytoma *n* = 6, anaplastic pleomorphic xanthoastrocytoma (PXA) *n* = 2, astroblastoma *n* = 1, neuroepithelial tumour *n* = 1] (G) Tumour mutation burden (TMB) of primary and recurrent specimens in comparison with brain tumours in patients with confirmed CMMRD (*n* = 130; data from IRRDC, https://replicationrepair.ca/; unpublished); the TMB was higher than that previously reported in the published literature for MMR‐proficient (MMRP) ependymomas (*n* = 70; *p* = 0.001; Mann–Whitney test [44]). (H) Comparison of variants detected in the primary specimen versus those at relapse. As compared to baseline (upper panel), both higher single nucleotide variant (SNV) and indel allele frequency were detected at relapse (lower panel). Primary (upper) and relapse (lower panel) sample WGS mutational (SBS) signatures showed the presence of COSMIC SBS 44 (13%) in primary and SBS 15 (12%), SBS 26 (13.5%) and SBS 20 (14.5%) at relapse confirming that both tumours were driven by DNA mismatch‐repair (MMR) deficiency. Other signatures detected in the primary included SBS 1 (spontaneous deamination of 5‐methylcytosine), 7c (UV exposure), 16, 37, 89 (unknown), and 92 (tobacco), and in the first recurrence, SBS 7c (UV exposure), 31 (platinum chemotherapy), 34, and 89 (unknown). (I) Evaluation of the tumour microenvironment using immunohistochemistry showed focal clusters of CD8 (+) T cells both at diagnosis and relapse. (J) Estimates of CD8 infiltration using the DIME score using methylation data as described by Safaei *et al* [22] showed higher CD8 T‐cell scores for the index patient as compared with MMR‐proficient ependymomas (*p* = 0.0087; Mann–Whitney test).

Meanwhile, paired whole genome sequencing (WGS) had been undertaken as part of the NHS‐England 100,000 Genomes Project (https://www.genomicsengland.co.uk/initiatives/100000-genomes-project). This displayed an excess of single nucleotide variants (SNVs) and indels in both the primary and recurrent samples, compared with MMR‐proficient paediatric brain tumours (Figure 2F). The tumour mutation burden (TMB) at diagnosis and relapse (1.93 and 5.09 non‐synonymous SNV per Mb, respectively) were higher than that reported in ependymomas in the published literature (Figure 2G). Comparative analyses further revealed that all classes of variants were not only elevated in the relapse compared with the primary diagnostic specimen but also showed higher allelic frequencies (Figure 2H). Although there was only minimal overlap (4%) of specific variants (Figure 2H), mutational signature (COSMIC V3.2; https://cancer.sanger.ac.uk/signatures/sbs/) analyses [17] demonstrated defective DNA mismatch‐repair signatures in both primary (13%) and relapse (40%) specimens, indicating that the clonal evolution and mutation accumulation was plausibly driven by DNA mismatch‐repair deficiency and related genomic instability. Putative driver variants that were exclusive to the relapse included truncating variants in *ARID1A* (p.Gln802SerfsTer15), *ASXL1* (p.Gly646TrpfsTer12) and *CIC* (p.Leu510ThrfsTer4, p.Leu1249ThrfsTer6) (Table S1). Though variants in *ASXL1* and *CIC* have been reported previously in posterior fossa ependymomas [18], all three variants resided at repetitive homopolymer loci, and it is unclear if they confer a selective advantage or are benign passenger mutations accumulating as a result of defective MMR. We did not detect any notable variants in *CXorf67* or the histone H3 genes [18, 19, 20], nor did we detect any *TP53* and *MAPK* pathway aberrations that we have previously described in gliomas arising in children with CMMRD [21].

The relatively high TMB and MSI also plausibly contributed to the high CD8+ T‐cell infiltration in the microenvironment, suggesting high immunogenicity. This was evident as multifocal areas of CD8 staining on immunohistochemistry (Figure 2I), as well as on estimation of CD8 T‐cell infiltration estimates using methylation studies performed as previously published [22], which demonstrated a higher score for our patient in comparison with MMR‐proficient ependymomas (Figure 2J). PD‐L1 was not expressed in either the primary or the relapse specimen.

In view of the elevated TMB, MSI and presence of T‐cells in the microenvironment in the previous biopsies, a decision was made following the second recurrence, to proceed with immune‐checkpoint‐inhibition using nivolumab [8], a human monoclonal antibody that targets and inhibits the programmed death‐1 (PD‐1) cluster of differentiation 279 (CD279) cell surface membrane receptor, on a compassionate basis. The patient currently remains clinically well on immunotherapy. Given the high risk of further malignancies, the patient continues to undergo close surveillance with clinical review, three‐monthly MRI head/spine and six‐monthly abdominal ultrasound, consistent with current CMMRD guidelines [23]. She is now 5 years of age and 44 months from initial presentation with ependymoma, 36 and 12 months from first and second localised recurrences, respectively.

We describe an unusual case of CMMRD manifesting at a very young age, with an ependymoma, a tumour not previously described with CMMRD. A recent study reported that 44% of cancers develop in the CNS, 27% in the gastrointestinal tract, 19% are haematological malignancies and 10% are other solid tumours [7, 24]. Other reported malignancies include endometrial, renal and urinary tract tumours, osteosarcoma, angiosarcoma and rhabdomyosarcomas [23, 25]. The most frequently reported cause is biallelic mutations in *PMS2* [24, 26]. Reported cases show a stronger prevalence of CNS malignancies in the presence of biallelic mutations in *PMS2* and *MSH6*, compared with *MLH1* and *MSH2* mutations [9]. The clinical features of CMMRD, as occurred in this case, often mimic neurofibromatosis with café‐au‐lait macules as the most common manifestation. Indeed, CMMRD is diagnosed in 0.4% of suspected sporadic neurofibromatosis, negative for pathogenic variants in *NF1* and *SPRED1* [27]. Impaired immunoglobulin class switch has also been reported in association with *PMS2* deficiency [28], resulting in mild immunodeficiency.

Timely diagnosis is crucial for patient management in CMMRD; however, due to lack of awareness and diagnostic difficulties, this is often delayed. The European Consortium ‘Care 4 CMMRD’ (C4MMRD) propose a clinical diagnostic protocol based on a three‐point scoring system, with all patients reaching the defined threshold further investigated by MSI analysis or immunohistochemistry [24]. However, our patient would not have been identified for CMMRD work‐up using this system. As we have shown, immunohistochemistry is a reliable and easy‐to‐perform screening test, with loss of protein expression in both neoplastic and normal cells highly concordant with the diagnosis. MMR deficiency is routinely assayed in all colorectal and endometrial cancers [29, 30], but at present, it is not recommended routinely for paediatric brain tumours. As this diagnosis has important clinical implications for both management of cancer and the family, and because sequencing is not routinely performed in all settings, we advocate screening for MMR‐deficiency using an inexpensive tool like immunohistochemistry in all children with CNS tumours, the utility of which has been demonstrated in previous reports [5, 9, 31, 32, 33]. This needs to be followed by genetic and functional testing if indicated, as was performed on our patient.

The diagnosis of CMMRD in our patient was followed by comprehensive genomic and immune analyses through international partnerships that confirmed that the ependymoma was indeed arising from DNA replication‐repair defects and was not a chance association, leading to important insights that impacted clinical decisions. The genomic instability and high TMB, in combination with the high tumour genomic MSI, plausibly led to neoantigen expression and the relative abundance of immune infiltrates [34]. The biopsy at recurrence, though demonstrating higher cell density and proliferation with higher mutation accumulation, still retained the PFA ependymoma subgroup affiliation on methylation, as previously reported in other relapsed ependymoma tumours [35, 36, 37]. This higher TMB at recurrence, in combination with a higher proliferative index, suggests that mutation accumulation in CMMRD is a continuous process that can increase with each cell division, and over time [38]. In this context, it is notable that the more commonly reported malignant gliomas in CMMRD, which are usually seen in older children, harboured higher TMB than was observed in our 17‐month‐old patient with ependymoma. Further, these gliomas can gain extreme TMB exceeding 100 mutations/Mb if they acquire second hits in *POLE* or *POLD1* [38], neither of which were detected in our patient. Importantly, the ongoing high mutation accumulation in our patient did impact the tumour immune microenvironment and provided a rationale for treatment with nivolumab at second recurrence [8]. Importantly, at this stage, other conventional therapeutic options were extremely limited and would have likely involved reirradiation [39], or early‐phase approaches without proven efficacy.

The diagnosis of CMMRD also led to the initiation of a surveillance protocol for second neoplasms in our patient, as these can develop in up to 50% of children with CMMRD [40], and if diagnosed early and treated appropriately, can impact patient survival [6]. Importantly, monoallelic mutations in any of the MMR genes characterise the autosomal dominant cancer syndrome, Lynch syndrome [41]. Targeted *PMS2* sequencing was performed on family members at risk. As a result of our patient's diagnosis, Lynch syndrome was identified molecularly in both her parents. In *PMS2*‐associated CMMRD, there is often no family history [26], as Lynch syndrome associated with *PMS2* mutations has a relatively low age‐dependent penetrance with a reported mean age of 52 years at presentation [42]. It is therefore not unusual for the parents to be unaffected. Due to this milder phenotype, routine screening for colorectal cancer in *PMS2‐*associated Lynch syndrome is recommended from the age of 35 years, in contrast with *MLH1/MSH2* mutations where screening is advised from the age of 25 years [43].

In summary, this report highlights a rare but important diagnosis of CMMRD within the context of a tumour type where it would not be expected (ependymoma). Given the significant management implications for the patient and at‐risk family members, and the simplicity and low cost of the immunohistochemical test, we propose that screening for CMMRD using immunohistochemistry should be strongly considered in all malignant paediatric CNS neoplasms, particularly as this can allow exploration of immune‐directed strategies for tumours which fail conventional therapies.

## CONFLICTS OF INTEREST

The authors declare no conflicts of interest.

## AUTHOR CONTRIBUTIONS

Study concept: MB, AD, HF, MJM; Methodology: MB, AD, PT, UT, KA, MJM; Data analysis and interpretation: MB, AD, HF, AL, SSR, LN, ABE, JC, JT, PT, UT, KA, MJM; Literature Review: MB, AD, MJM; Figure acquisition/production: AD, PP, KA, SSR, ABE, JT, PT, MJM; Clinical input: IJ, PP, NT, RGG, CH, KA, UT, MJM; Manuscript writing: MB, AD, PT, MJM; Manuscript revision and approval: MB, AD, HF, AL, SSR, LN, ABE, JC, VB, IJ, PP, NT, RGG, CH, JT, PT, UT, KA, MJM.

## ETHICS STATEMENT

All procedures performed involving human participants were in accordance with the ethical standards of the institutional and national research committee and with the 1964 Helsinki declaration and its later amendments or comparable ethical standards. Informed consent was obtained from the parents.

## CONSENT TO PUBLISH

Parental consent was obtained.

### PEER REVIEW

The peer review history for this article is available at https://publons.com/publon/10.1111/nan.12862.

## Supporting information


**Data S1.** Supporting Information

## Data Availability

The data that supports the findings of this study are available in the supplementary material of this article.

## References

[1] Abedalthagafi M . Constitutional mismatch repair‐deficiency: current problems and emerging therapeutic strategies. Oncotarget. 2018;9(83):35458‐35469. 30459937 10.18632/oncotarget.26249 PMC6226037

[2] Ricciardone MD , Ozçelik T , Cevher B , et al. Human MLH1 deficiency predisposes to haematological malignancy and neurofibromatosis type 1. Cancer Res. 1999;59(2):290‐293. 9927033

[3] Wang Q , Lasset C , Desseigne F , et al. Neurofibromatosis and early onset of cancers in hMLH1‐deficient children. Cancer Res. 1999;59(2):294‐297. 9927034

[4] Amayiri N , Tabori U , Campbell B , et al. High frequency of mismatch repair deficiency among paediatric high grade gliomas in Jordan. Int J Cancer. 2016;138(2):380‐385. doi:10.1002/ijc.29724 26293621

[5] Alphones S , Chatterjee U , Singh A , et al. Immunohistochemical screening for mismatch repair protein deficiency in paediatric high‐grade gliomas—institutional experience and review of literature. Childs Nerv Syst. 2021;37(8):2521‐2530. doi:10.1007/s00381-021-05229-1 34097097

[6] Durno C , Ercan A , Bianchi V , et al. Survival benefit for individuals with constitutional mismatch repair deficiency undergoing surveillance. J Clin Oncol. 2021;39(25):2779‐2790. doi:10.1200/JCO.20.02636 33945292 PMC8407605

[7] Wimmer K , Etzler J . Constitutional mismatch repair‐deficiency syndrome: have we so far seen only the tip of an iceberg? Human Genet. 2008;124(2):105‐122. 18709565 10.1007/s00439-008-0542-4

[8] Das A , Sudhaman S , Morgenstern D , et al. Genomic predictors of response to PD‐1 inhibition in children with germline DNA replication repair deficiency. Nat Med. 2022;28(1):125‐135. doi:10.1038/s41591-021-01581-6 34992263 PMC8799468

[9] Guerrini‐Rousseau L , Varlet P , Colas C , et al. Constitutional mismatch repair deficiency–associated brain tumours: report from the European C4CMMRD consortium. Neuro‐Oncol Adv. 2019;1(1):vdz033. 10.1093/noajnl/vdz033 PMC7212899 32642664

[10] Baroni LV , Sundaresan L , Heled A , et al. Ultra high‐risk PFA ependymoma is characterized by loss of chromosome 6q. Neuro Oncol. 2021;23(8):1360‐1370. doi:10.1093/neuonc/noab034 33580238 PMC8328032

[11] Truninger K , Menigatti M , Luz J , et al. Immunohistochemical analysis reveals high frequency of PMS2 defects in colorectal cancer. Gastroenterology. 2005;128(5):1160‐1171. doi:10.1053/j.gastro.2005.01.056 15887099

[12] Yurgelun MB , Allen B , Kaldate RR , et al. Identification of a variety of mutations in cancer predisposition genes in patients with suspected lynch syndrome. Gastroenterology. 2015;149(3):604‐613. doi:10.1053/j.gastro.2015.05.006 25980754 PMC4550537

[13] Hildreth A , Valasek MA , Thung I , et al. Biallelic mismatch repair deficiency in an adolescent female. Case Rep Genet. 2018;2018:e8657823. 10.1155/2018/8657823 PMC6092986 30155321

[14] Cheyuo C , Radwan W , Ahn J , Gyure K , Qaiser R , Tomboc P . Biallelic PMS2 mutation and heterozygous DICER1 mutation presenting as constitutional mismatch repair deficiency with corpus callosum agenesis: case report and review of literature. J Pediatr Hematol Oncol. 2017;39(7):e381‐e387. doi:10.1097/MPH.0000000000000863 28562508

[15] Lukas C , Crenshaw M , Gonzalez‐Gomez I , et al. Compound heterozygous mutation of the PMS2 gene in an infant with constitutional mismatch repair deficiency and medulloblastoma. Neuro‐Oncol. 2018;20(Supplement_2):i127.

[16] Bodo S , Colas C , Buhard O , et al. Diagnosis of constitutional mismatch repair‐deficiency syndrome based on microsatellite instability and lymphocyte tolerance to methylating agents. Gastroenterology. 2015;149(4):1017‐1029. doi:10.1053/j.gastro.2015.06.013 26116798

[17] Alexandrov L , Kim J , Haradhvala N , et al. The repertoire of mutational signatures in human cancer. Nature. 2020;578(7793):94‐101. doi:10.1038/s41586-020-1943-3 32025018 PMC7054213

[18] de Carvalho Corrêa DC , Tesser‐Gamba F , Oliveira ID , et al. Molecular profiling of pediatric and adolescent ependymomas: identification of genetic variants using a next‐generation sequencing panel. J Neurooncol. 2021;155(1):13‐23. doi:10.1007/s11060-021-03848-x 34570300

[19] Pajtler KW , Wen J , Sill M , et al. Molecular heterogeneity and CXorf67 alterations in posterior fossa group A (PFA) ependymomas. Acta Neuropathol. 2018;136(2):211‐226. doi:10.1007/s00401-018-1877-0 29909548 PMC6105278

[20] Ryall S , Guzman M , Elbabaa SK , et al. H3 K27M mutations are extremely rare in posterior fossa group A ependymoma. Childs Nerv Syst. 2017;33(7):1047‐1051. doi:10.1007/s00381-017-3481-3 28623522

[21] Campbell BB , Galati MA , Stone SC , et al. Mutations in the RAS/MAPK pathway drive replication repair‐deficient hypermutated tumours and confer sensitivity to MEK inhibition. Cancer Discov. 2021;11(6):1454‐1467. doi:10.1158/2159-8290.CD-20-1050 33563663 PMC8406556

[22] Safaei S , Mohme M , Niesen J , Schüller U , Bockmayr M . DIMEimmune: robust estimation of infiltrating lymphocytes in CNS tumours from DNA methylation profiles. Oncoimmunology. 2021;10(1):1932365. 34235002 10.1080/2162402X.2021.1932365 PMC8216185

[23] Tabori U , Hansford JR , Achatz MI , et al. Clinical management and tumour surveillance recommendations of inherited mismatch repair deficiency in childhood. Clin Cancer Res. 2017;23(11):e32‐e37. doi:10.1158/1078-0432.CCR-17-0574 28572265

[24] Wimmer K , Kratz CP , Vasen HF , et al. Diagnostic criteria for constitutional mismatch repair deficiency syndrome: suggestions of the European consortium ‘Care for CMMRD’ (C4CMMRD). J Med Genet. 2014;51(6):355‐365. doi:10.1136/jmedgenet-2014-102284 24737826

[25] Daou B , Zanello M , Varlet P , et al. An unusual case of constitutional mismatch repair deficiency syndrome with anaplastic ganglioglioma, colonic adenocarcinoma, osteosarcoma, acute myeloid leukemia, and signs of neurofibromatosis type 1: case report. Neurosurgery. 2015;77(1):E145‐E152. doi:10.1227/NEU.0000000000000754 25850602

[26] Sijmons RH , Hofstra RMW . Review: clinical aspects of hereditary DNA mismatch repair gene mutations. DNA Repair. 2016;38:155‐162. 26746812 10.1016/j.dnarep.2015.11.018

[27] Perez‐Valencia JA , Gallon R , Chen Y , et al. Constitutional mismatch repair deficiency is the diagnosis in 0.41% of pathogenic NF1/SPRED1 variant negative children suspected of sporadic neurofibromatosis type 1. Genet Med. 2020;22(12):2081‐2088. doi:10.1038/s41436-020-0925-z 32773772 PMC7708300

[28] Péron S , Metin A , Gardès P , et al. Human PMS2 deficiency is associated with impaired immunoglobulin class switch recombination. J Exp Med. 2008;205(11):2465‐2472. doi:10.1084/jem.20080789 18824584 PMC2571921

[29] National Institute for Health and Care Excellence . Testing strategies for Lynch syndrome in people with endometrial cancer. 2020 [NICE Diagnostic Guidance 42]. Website. https://www.nice.org.uk/guidance/dg42 Accessed 27th April 2022.

[30] National Institute for Health and Care Excellence . Molecular testing strategies for Lynch syndrome in people with colorectal cancer. 2017 [NICE Diagnostic Guidance 27]. Website. https://www.nice.org.uk/guidance/dg27 Accessed 27th April 2022.

[31] Carrato C , Sanz C , Muñoz‐Mármol AM , et al. The challenge of diagnosing constitutional mismatch repair deficiency syndrome in brain malignancies from young individuals. Int J Mol Sci. 2021;22(9):4629. 33924881 10.3390/ijms22094629 PMC8124255

[32] Tan S , Wu X , Wang A , Ying L . Diagnostic challenges in a CMMRD patient with a novel mutation in the PMS2 gene: a case report. BMC Med Gen. 2021;14(1):184. 10.1186/s12920-021-01031-9 PMC8274000 34247610

[33] Mishra AK , Achari RB , Zameer L , et al. Germline biallelic mismatch repair deficiency in childhood glioblastoma and implications for clinical management. Neurol India. 2022;70(2):772‐774. doi:10.4103/0028-3886.344608 35532657

[34] Griesinger A , Ritzmann T , Lourdusamy A , et al. EPEN‐23. A computational analysis of the tumour immune microenvironment in paediatric ependymoma. Neuro Oncol. 2020;22(Supplement_3):iii312.

[35] Yang D , Holsten T , Börnigen D , et al. Ependymoma relapse goes along with a relatively stable epigenome, but a severely altered tumour morphology. Brain Pathol. 2021;31(1):33‐44. doi:10.1111/bpa.12875 32633004 PMC8018105

[36] Pajtler KW , Witt H , Sill M , et al. Molecular classification of ependymal tumours across all CNS compartments, histopathological grades, and age groups. Cancer Cell. 2015;27(5):728‐743. doi:10.1016/j.ccell.2015.04.002 25965575 PMC4712639

[37] Dodgshun AJ , Fukuoka K , Edwards M , et al. Germline‐driven replication repair‐deficient high‐grade gliomas exhibit unique hypomethylation patterns. Acta Neuropathol. 2020;140(5):765‐776. doi:10.1007/s00401-020-02209-8 32895736

[38] Shlien A , Campbell B , de Borja R , et al. Combined hereditary and somatic mutations of replication error repair genes result in rapid onset of ultra‐hypermutated cancers. Nat Genet. 2015;47(3):257‐262. doi:10.1038/ng.3202 25642631

[39] Tsang DS , Burghen E , Klimo P , Boop FA , Ellison DW , Merchant TE . Outcomes after reirradiation for recurrent pediatric intracranial ependymoma. Int J Radiat Oncol Biol Phys. 2018;100(2):507‐515. doi:10.1016/j.ijrobp.2017.10.002 29229328

[40] Henderson JJ , Das A , Morgenstern DA , et al. Immune checkpoint inhibition as single therapy for synchronous cancers exhibiting hypermutation: an IRRDC study. JCO Precis Oncol. 2022;6(6):e2100286. 35235414 10.1200/PO.21.00286 PMC8906457

[41] Cerretelli G , Ager A , Arends MJ , Frayling IM . Molecular pathology of Lynch syndrome. J Pathol. 2020;250(5):518‐531. doi:10.1002/path.5422 32141610

[42] Ten Broeke SW , Brohet RM , Tops CM , et al. Lynch syndrome caused by germline PMS2 mutations: delineating the cancer risk. J Clin Oncol. 2015;33(5):319‐325. 25512458 10.1200/JCO.2014.57.8088

[43] Monahan KJ , Bradshaw N , Dolwani S , et al. Guidelines for the management of hereditary colorectal cancer from the British Society of Gastroenterology (BSG)/Association of Coloproctology of Great Britain and Ireland (ACPGBI)/United Kingdom cancer genetics group (UKCGG). Gut. 2020;69(3):411‐444. doi:10.1136/gutjnl-2019-319915 31780574 PMC7034349

[44] Gröbner SN , Worst BC , Weischenfeldt J , et al. The landscape of genomic alterations across childhood cancers. Nature. 2018;555(7696):321‐327. doi:10.1038/nature25480 29489754

